# The complete chloroplast genome and phylogenetic analysis of *Artocarpus nitidus* subsp. *lingnanensis* (Moraceae)

**DOI:** 10.1080/23802359.2026.2642520

**Published:** 2026-03-13

**Authors:** Qianying Qiu, Xuemei Chen, Wei Lin

**Affiliations:** ^a^ Academy of Fine Arts and Design, Huaihua University, Huaihua, China; ^b^ Science and Technology Department, Foshan Institute of Forestry (Foshan Botanical Garden), Foshan, China

**Keywords:** Complete chloroplast genome, *Artocarpus nitidus* subsp. *lingnanensis*, phylogenetic analysis

## Abstract

*Artocarpus nitidus* subsp. *lingnanensis* is an evergreen tree species bearing sour-sweet fruits. Although its economic and medicinal potential has been indicated by some phytochemical studies, the phylogenetic position of this species remains unclear. To address this, we sequenced and assembled its complete chloroplast genome for the first time. The genome with a total length of 161,009 bp and a GC content of 35.79%, displays the conventional quadripartite organization comprising four segments: an 89,552 bp large single-copy (LSC) region, a 20,093 bp small single-copy (SSC) region, and two 25,682 bp inverted repeat (IR) regions. Annotation identified 132 genes, encompassing 87 protein-coding genes, 37 tRNAs, and 8 rRNAs. Phylogenetic analysis revealed that *A. nitidus* subsp. *lingnanensis* forms a distinct clade with its closest relatives, *A. petelotii*, *A. tonkinensis*, *A. hypargyreus*, and *A. gomezianus*. This study provides the first complete chloroplast genomic resource for *A. nitidus* subsp. *lingnanensis* and offers valuable data for future research on genetic diversity and utilization.

## Introduction

*Artocarpus nitidus* subsp. *lingnanensis* (Merr.) F.M. Jarrett 1960 (Moraceae family) is an evergreen tree species can grow up to 17 meters in height (Zhang and Wu 1998). It is native to southern China, and also cultivated in parts of Southeast Asia. The bark of *A. nitidus* subsp. *lingnanensis* is dark and its timber is dense and durable, making it suitable for construction and furniture. Its edible fruits possess orange flesh with a sweet-sour taste and can be processed into many products (Luo et al. 2025). Fruit contains many active substances that can be used to treat diseases (Luo et al. 2018). Owing to its broad, dense canopy, it is also planted as an ornamental street tree.

**Figure 1.**
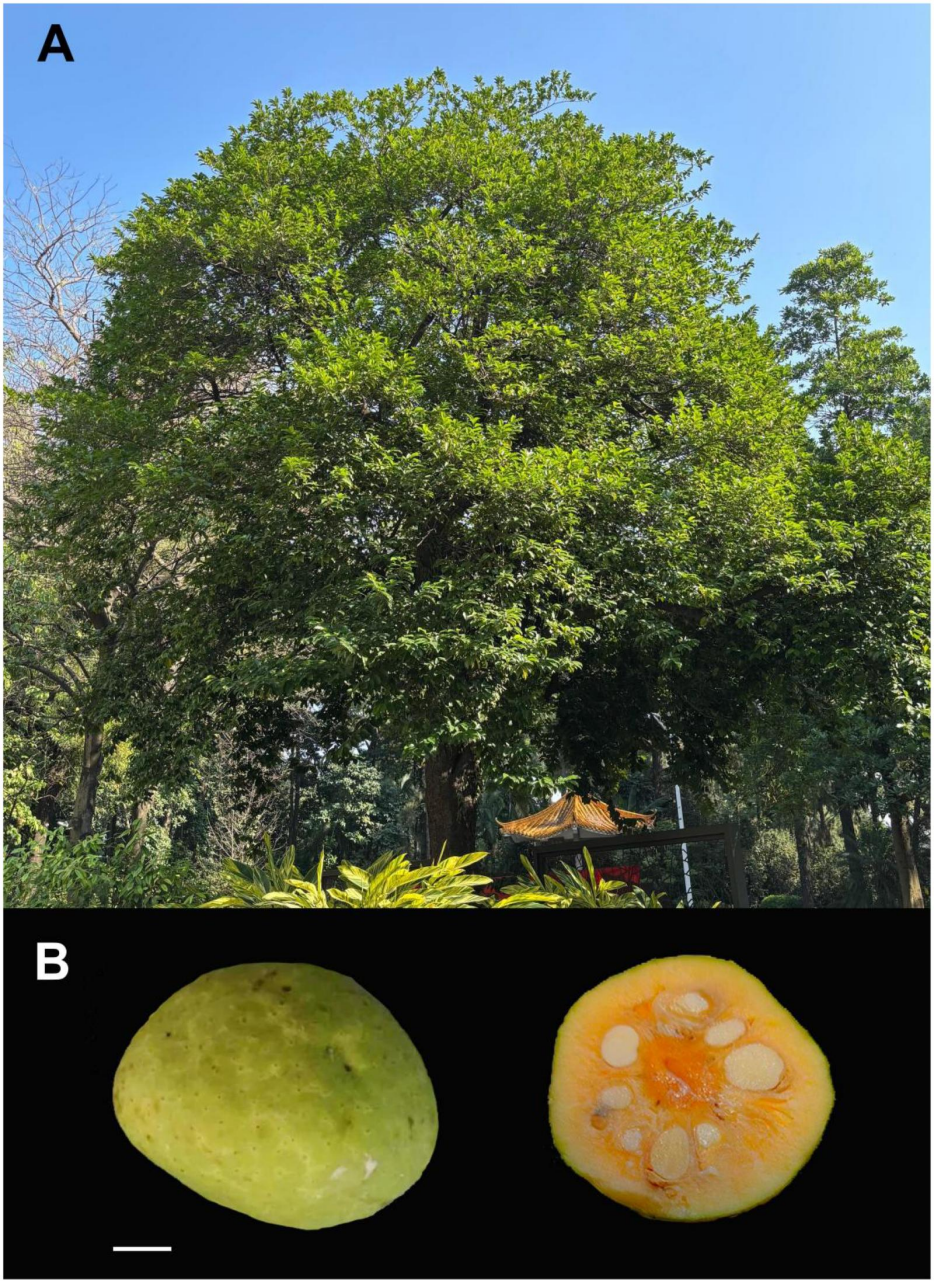
Morphology of *A. nitidus* subsp. *lingnanensis*. (A) The whole plant, 7–8 m tall, showing the evergreen canopy and the brown bark. (B) The aggregate fruit and its cross section, revealing the green rind and orange pulp. Scale bar represent 1 cm. Photographed by Xuemei Chen at Foshan Botanical Garden, Guangdong Province, China.

Despite its various uses, taxonomic and phylogenetic knowledge of *A. nitidus* subsp. *lingnanensis* remains limited. In particular, the characteristics of its chloroplast genome have not been previously reported. The unclear species boundaries and phylogenetic placement hinder accurate identification, leading to potential mislabeling in germplasm collections and obscuring conservation priorities. Furthermore, the lack of genomic resources, prevents the development of reliable molecular markers for population genetics studies and breeding programs. Chloroplast genomes are valuable for understanding plant phylogeny and supporting genetic studies, as they contain conserved genes related to photosynthesis and other essential functions (Lestari et al. 2024). To elucidate its phylogenetic relationships, we generated and analyzed the first complete chloroplast genome of *A. nitidus* subsp. *lingnanensis*. These findings provide new genomic resources that will facilitate further research on this species and its relatives (Figure 1).

## Materials and methods

Fresh leaf samples of *A. nitidus* subsp. *lingnanensis* were obtained from the Foshan Botanical Garden in Guangdong Province, China (23°11′23″N, 113°00′52″E). The voucher specimen was deposited in the herbarium of Foshan Botanical Garden (Foshan, China, Xueyi Tian, tianxueyi11@sina.com) under the number FBA-1. Total genomic DNA was extracted from approximately 100 mg of fresh leaf tissue using a modified CTAB method (Doyle and Doyle 1987). The concentration and quality of the extracted DNA were assessed with a NanoDrop 2000 spectrophotometer and agarose gel electrophoresis. A paired-end sequencing library was constructed with the NEBNext® Ultra™ II DNA Library Prep Kit (Illumina, USA) following the manufacturer’s protocol and sequenced on an Illumina novaseq X plus platform to generate 150-bp paired-end reads. Raw sequencing reads were quality-filtered using fastp (v0.23.4) (Li et al. 2025). The chloroplast genome was then de novo assembled with GetOrganelle (v1.7.5.0) with the k-mer values of 55, 87 and 121 (Jin et al. 2020). Annotation was performed using CPGAVAS2 and GeSeq (Tillich et al. 2017), with tRNA genes further verified by tRNAscan-SE. A circular map of the chloroplast genome was generated with OGDraw (Lohse et al. 2007). The cis/trans-splicing genes maps were drawn by CPGView software (Liu et al. 2023). The complete chloroplast genome sequence was deposited in GenBank under the accession number PX696983.

Chloroplast genome sequences were aligned using MAFFT (v7.505) with the “auto” strategy (Katoh et al. 2019), following the removal of one copy of the inverted repeats to eliminate redundant sites. The alignment comprised 17 sequences in total: 12 *Artocarpus* genomes downloaded from NCBI, plus the newly sequenced *A. nitidus* subsp. *lingnanensis*. The alignment was trimmed with trimAI (v1.4.rev15). A maximum likelihood phylogeny was reconstructed in RAxML (v8.2.10) under the GTRGAMMA model, which was previously determined to be the best-fit model by jModelTest (v2.1.10) using the Bayesian information criterion. The analysis, which included the designated outgroup *Morus alba* (NCBI accession: OP380683.1), employed 1000 rapid bootstrap replicates for branch support estimation (Stamatakis 2014).

## Results

**Figure 2.**
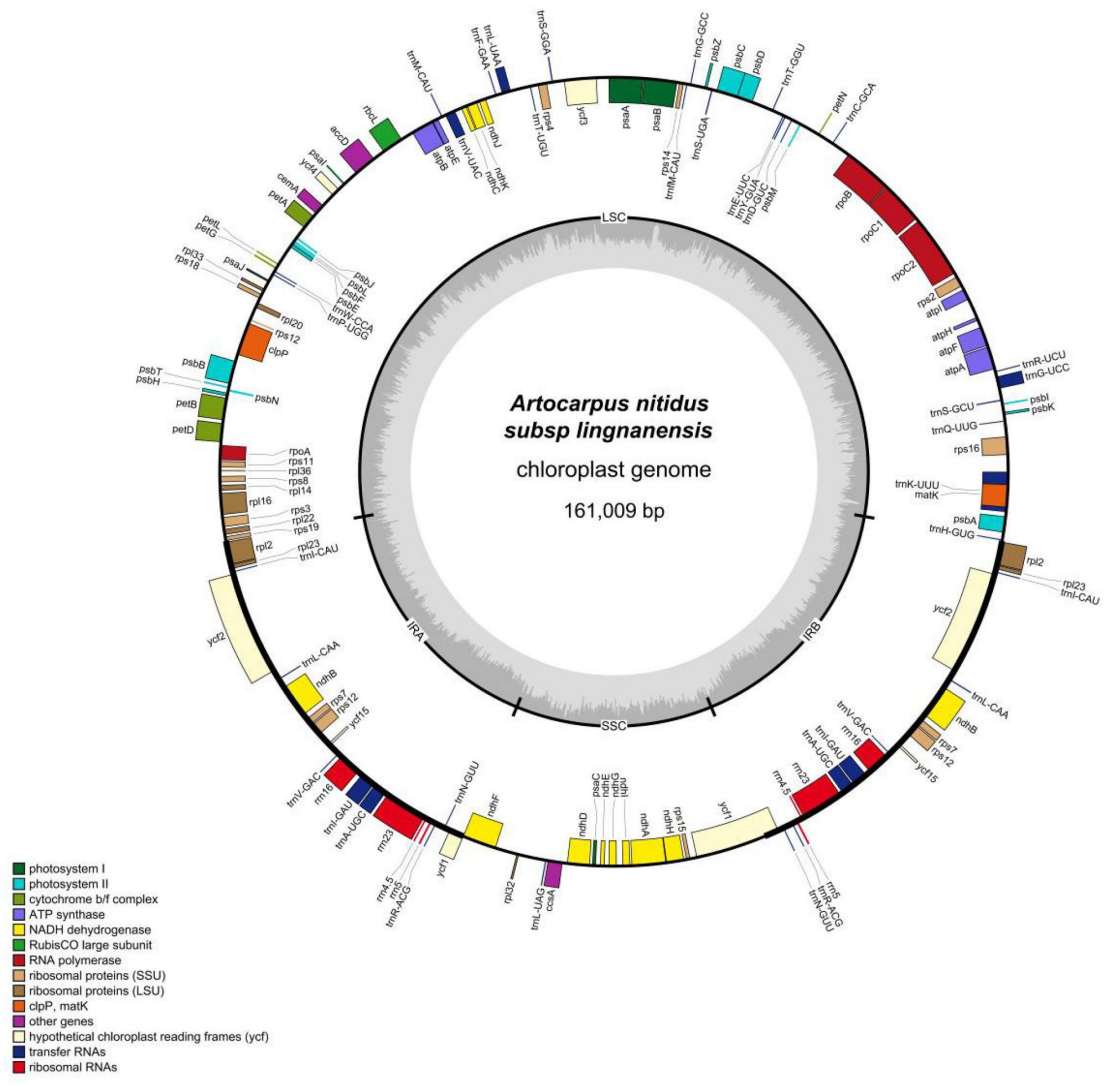
Circular map of the *A. nitidus* subsp. *lingnanensis* chloroplast genome. Genes drawn inside the circle are transcribed clockwise, while those outside are transcribed counterclockwise. The inner ring denotes the borders of the large single-copy (LSC), small single-copy (SSC), and inverted repeat (IRA/IRB) regions. Genes are color-coded by functional category, as shown in the legend. The innermost gray ring illustrates the GC content.

Featuring the conserved quadripartite structure (Figure 2), the chloroplast genome of *A. nitidu*s subsp. *lingnanensis* comprises an 89,552 bp LSC, a 20,093 bp SSC, and two 25,682 bp IRs, with a total length of 161,009 bp, with an average sequencing depth of 824.1× (Figure S1). The overall GC content is 35.79%. A total of 132 genes are annotated, including 87 protein-coding genes, 37 tRNAs, and 8 rRNAs (Tables S1). Thirteen cis-splicing genes and the trans-splicing gene rps12 were detected (Figures S2).

**Figure 3.**
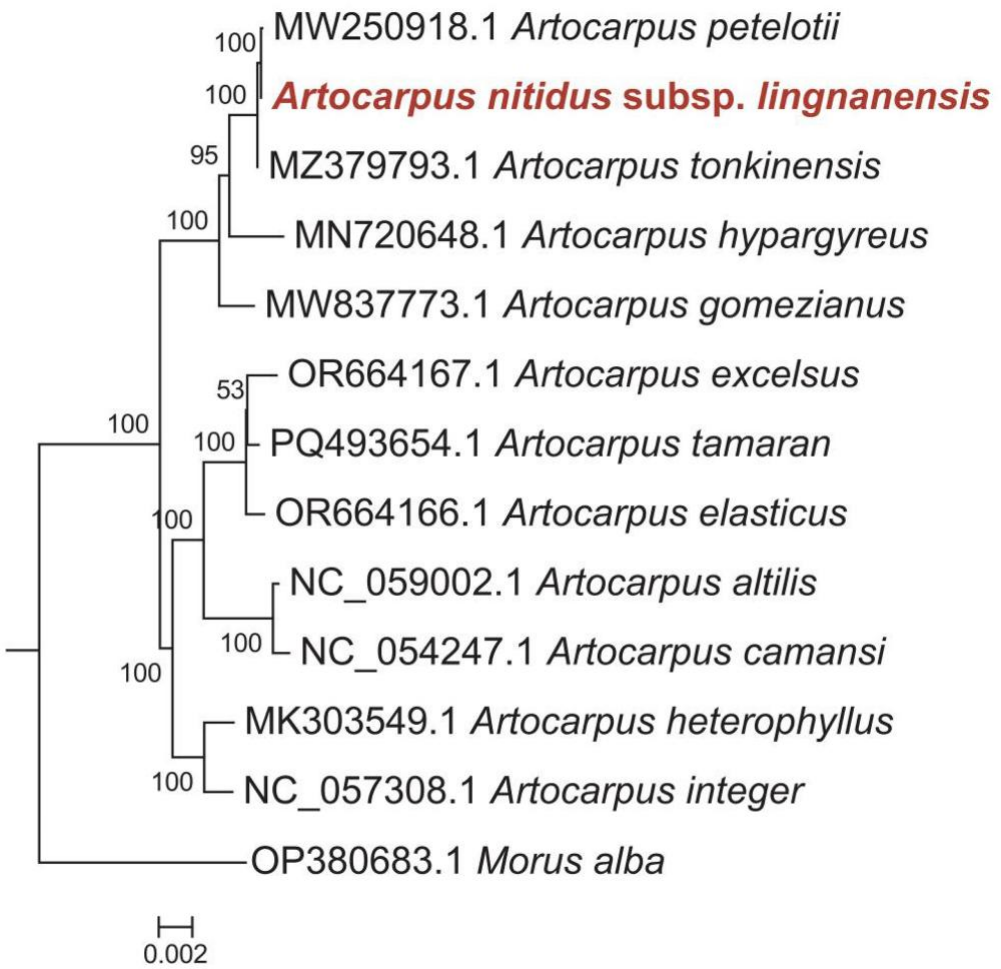
Phylogenetic tree constructed from complete chloroplast genomes to determine the evolutionary position of *A. nitidus* subsp. *lingnanensis* (highlighted). Bootstrap support values (>50%) are shown on the branches. The sequences used were as followed: *A. nitidus* subsp. *lingnanensis* (PX696983, this study); *Artocarpus tonkinensis*, MZ379793.1; *Artocarpus heterophyllus*, MK303549.1; *Artocarpus hypargyreus*, MN720648.1; *Artocarpus petelotii*, MW250918.1; *Artocarpus gomezianus*, MW837773.1; *Artocarpus altilis*, NC059002.1; *Artocarpus camansi*, NC054247.1; *Artocarpus tamaran*, PQ493654.1; *Artocarpus elasticus*, OR664166.1; *Artocarpus integer*, MT900597.1; *Artocarpus excelsus*, OR664167.1; *Morus alba*, OP380683.1.

Using the complete chloroplast genome sequences, a maximum likelihood (ML) tree was reconstructed to clarify the phylogenetic placement of *A. nitidus* subsp. *lingnanensis* within *Artocarpus*. Our dataset included the newly sequenced genome along with 11 additional *Artocarpus* species. *M. alba*, a closely related genus within Moraceae, was used as the outgroup for rooting the phylogenetic tree. As depicted in Figure 3, the first clade including *A. nitidus* subsp. *lingnanensis, A. petelotii*, *A. tonkinensis*, *A. hypargyreus* and *A. gomezianus,* among them *A. nitidus* subsp. *lingnanensis* and *A. petelotii* are closely related, with a bootstrap support rate of 100%. The second clade contains 7 *Artocarpus* species, including *A. excelsus*, *A. tamaran*, *A. elasticus*, *A. camansi*, *A. altilis*, *A. integer* and *A. heterophyllus*. And the outgroup clustered into a distinct branch.

## Discussion and conclusion

Plants of the genus *Artocarpus* are tall trees valued for their edible fruit, high quality timber, and therapeutic properties, which give them significant economic importance (Chaurasia and Pandey 2023). However, chloroplast genome data are available for only a few species in this genus, limiting our understanding of its evolution and taxonomy. For the first time, the complete chloroplast genome of *A. nitidus* subsp. *lingnanensis* has been assembled and annotated. The resulting sequence displays the common quadripartite circular organization, which includes one large single-copy (LSC), one small single-copy (SSC), and two inverted repeat (IR) regions. Its size (161,009 bp), GC content (35.79%), and gene repertoire (132 genes) are highly conserved and fall within the range reported for other *Artocarpus* species (Chen and Liu 2021), which reinforces the structural stability of chloroplast genomes in this genus.

Phylogenetic analysis placed the five *Artocarpus* species within a single, strongly supported clade (bootstrap value > 95%), with *A. nitidus* subsp. *lingnanensis* being most closely related to *A. petelotii*. Despite this close genetic affinity, the two species can be easily distinguished by several morphological traits. *A. petelotii* has papery leaves covered with reddish-brown pubescence and features bears dense, prominent veins. In contrast, leaves of *A. nitidus* subsp. *lingnanensis* are glabrous with inconspicuous reticulate venation. Furthermore, *A. petelotii* exhibits longer inflorescences and petioles compared to those of *A. nitidus* subsp. *lingnanensis*. These morphological differences highlight the importance of integrating molecular and morphological data for accurate species delimitation within this complex group. The consistency between our phylogenetic results and those from previous chloroplast genome studies improves the resolution of species relationships within *Artocarpus* (Ho et al. 2025). The chloroplast genome of *A. nitidus* subsp. *lingnanensis* assembled in this study will facilitate future phylogenetic, evolutionary, and molecular identification research within the genus *Artocarpus*.

## Supplementary Material

Revised manuscript clean.docx

Figure S1.jpg

Table S1.xlsx

Figure S2.jpg

## Data Availability

The genome sequence data that support the findings of this study are openly available in GenBank of NCBI at [https://www.ncbi.nlm.nih.gov] under accession number PX696983. The associated BioProject, SRA, and Bio-Sample numbers are PRJNA1380063, SRR36419247 and SAMN53900006, respectively.
