# Supplementary figures and images for: The complete chloroplast genome and phylogenetic analysis of Artocarpus nitidus subsp. lingnanensis (Moraceae)
Source: Mitochondrial DNA B Resour. 2026 Mar 13;11(4):541–5. doi: 10.1080/23802359.2026.2642520 (PMC12990266; doi:10.1080/23802359.2026.2642520)

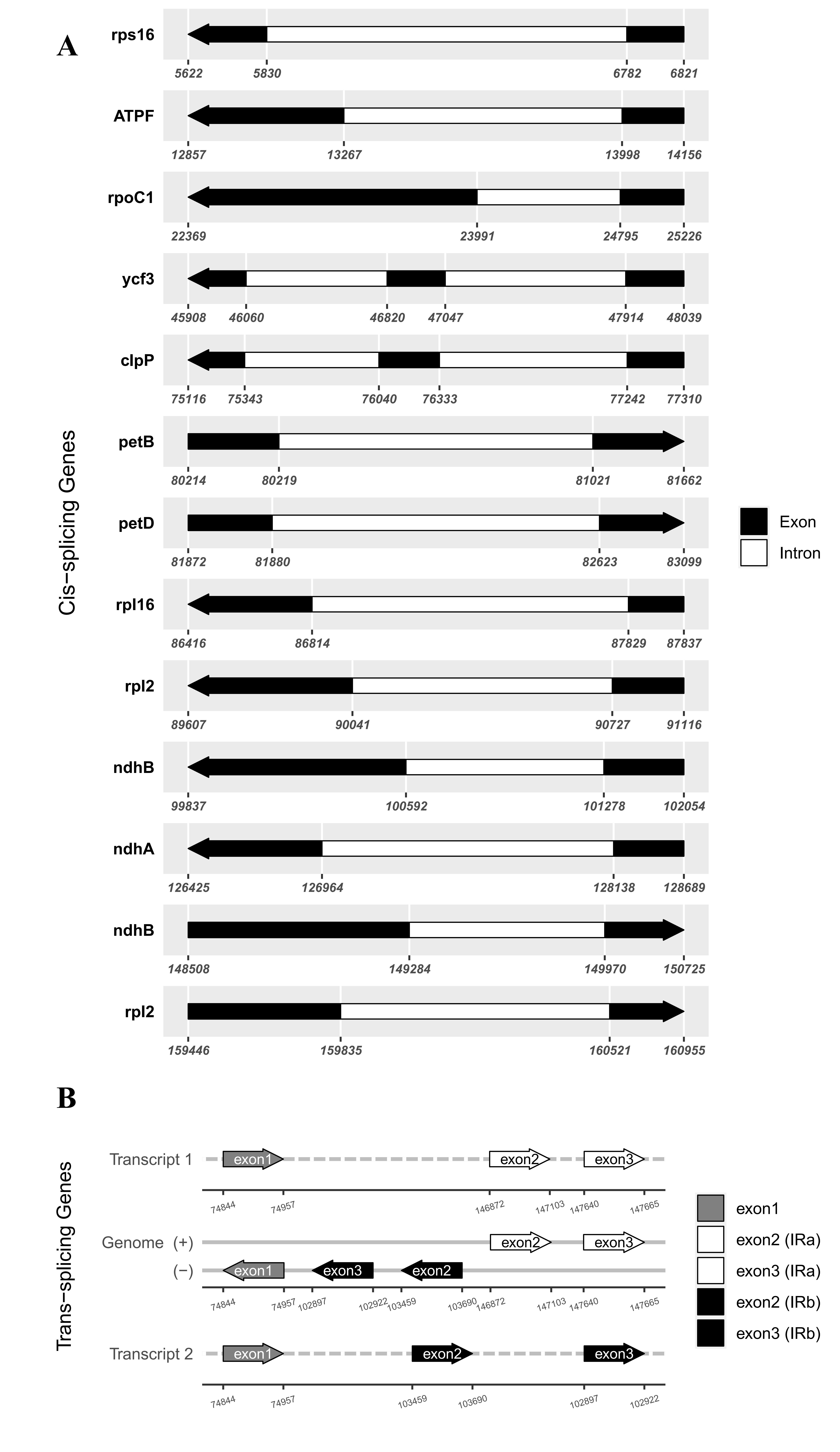

Supplement: Figure S2.jpg [file TMDN_A_2642520_SM5418.jpg]
